# Supplementary material for: Enzyme mechanistic studies of NMA1982, a protein tyrosine phosphatase and potential virulence factor in Neisseria meningitidis
Source: Sci Rep. 2023 Dec 12;13:22015. doi: 10.1038/s41598-023-49561-9 (PMC10716126; doi:10.1038/s41598-023-49561-9)
Supplement: Supplementary file 1 — Supplementary Figures. [file 41598_2023_49561_MOESM1_ESM.pdf]

Supplementary Information

Enzyme Mechanistic Studies of NMA1982, a Protein Tyrosine Phosphatase and Potential Virulence Factor in *Neisseria meningitidis*

Shuangding Wu[1], Mathieu Coureuil[2,3], Xavier Nassif[2,3], and Lutz Tautz[1]*

[1]NCI-Designated Cancer Center, Sanford Burnham Prebys Medical Discovery Institute, 10901 N Torrey Pines Rd, La Jolla, CA 92037, USA

[2]Université Paris Cité, UFR de Médecine, 15 Rue de l'École de Médecine, 75006 Paris, France

[3]Institut Necker Enfants-Malades, Inserm U1151, CNRS UMR 8253, 160 Rue de Vaugirard, 75015 Paris, France

* Corresponding author

E-mail: tautz@sbpdiscovery.org

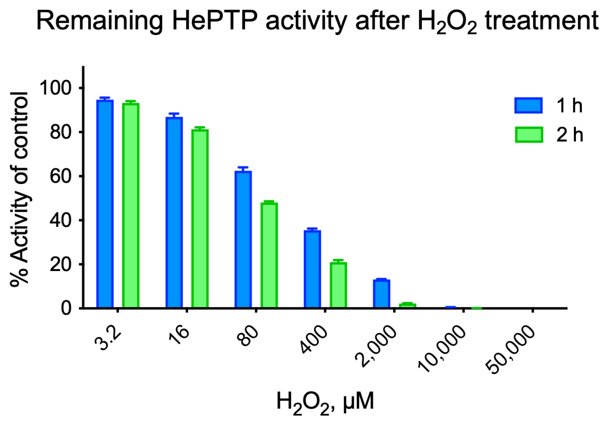

Supplementary Figure S1. Remaining HePTP activity in response to treatment with various concentrations of hydrogen peroxide for 1 or 2 h. The assay was performed similarly as described for NMA1982.

```
NG01376|ID:629062|          MAILKLDEHLYISPQLTKADAEQIAQLGIKTVICNRPDREEESQPDFAQIKQWLEQAGVT 60
NMCC_1640|ID:2722073|       MAILKLDEHLYISPQLTKADAEQIAQLGIKTVICNRPDREEESQPDFAQIKQWLEQAGVT 60
NMA1982|ID:135944|          MAILKLDEHLYISPQLTKADAEQIAQLGIKTVICNRPDREEESQPDFAQIKQWLEQAGVT 60
NMV_0640|ID:2191602|        MAILKLDEHLYISPQLTKADAEQIAQLGIKTVICNRPDREEESQPDFAQIKQWLEQAGVT 60
NMC1647|ID:1956222|         MAILKLDEHLYISPQLTKADAEQIAQLGIKTVICNRPDREEESQPDFAQIKQWLEQAGVT 60
NMB1727|ID:2018710|         MAILKLDEHLYISPQLTKADAEQIAQLGIKTVICNRPDREEESQPDFAQIKQWLEQAGVT 60
NGK_1613|ID:3088043|        MAILKLDEHLYISPQLTKADAEQIAQLGIKTVICNRPDREEESQPDFAQIKQWLEQAGVT 60
                            ************************************************************

NG01376|ID:629062|          GFHHQPVTARDIQKHDVETFRQLIGQAEYPILAYCRTGTRCSLLWGFRRAAEGMPVDEII 120
NMCC_1640|ID:2722073|       GFHHQPVTARDIQKHDVETFRQLIEQAEYPVLAYCRTGTRCSLLWGFRRAAEGMPVDEII 120
NMA1982|ID:135944|          GFHHQPVTARDIQKHDVETFRQLIGQAESPVLAYCRTGTRCSLLWGFRRAAEGMPVDEII 120
NMV_0640|ID:2191602|        GFHHQPVTARDIQKHDVETFRQLIGQAEYPVLAYCRTGTRCSLLWGFRRAAEGMPVDEII 120
NMC1647|ID:1956222|         GFHHQPVTARDIQKHDVETFRQLIGQAEYPVLAYCRTGTRCSLLWGFRRAAEGMPVDEII 120
NMB1727|ID:2018710|         GFHHQPVTARDIQKHDVETFRQLIGQAEYPVLAYCRTGTRCSLLWGFRRAAEGMPVDEII 120
NGK_1613|ID:3088043|        GFHHQPVTARDIQKHDVETFRQLIGQAEYPVLAYCRTGTRCSLLWGFRRAAEGMPVDEII 120
                            ************************ *** *:*****************************

NG01376|ID:629062|          RRAQAAGVNLENFRERLDNARV*      142
NMCC_1640|ID:2722073|       RRAQAAGVNLENFRERLDNARV*      142
NMA1982|ID:135944|          RRAQAAGVNLENFRERLDNARV*      142
NMV_0640|ID:2191602|        RRAQAAGVNLENFRERLDNARV*      142
NMC1647|ID:1956222|         RRAQAAGVNLENFRERLDNARV*      142
NMB1727|ID:2018710|         RRAQAAGVNLENFRERLDNARV*      142
NGK_1613|ID:3088043|        RRAQAAGVNLENFRERLDNARV*      142
                            **********************
```

Supplementary Figure S2. Clustal Omega Alignment of NMA1982 orthologs in various *Neisseria meningitidis* and *Neisseria gonorrhoeae* strains. DNA sequences were obtained from https://mage.genoscope.cns.fr/microscope/home/index.php. Start codons were confirmed using RBS Calculator (https://salislab.net/).

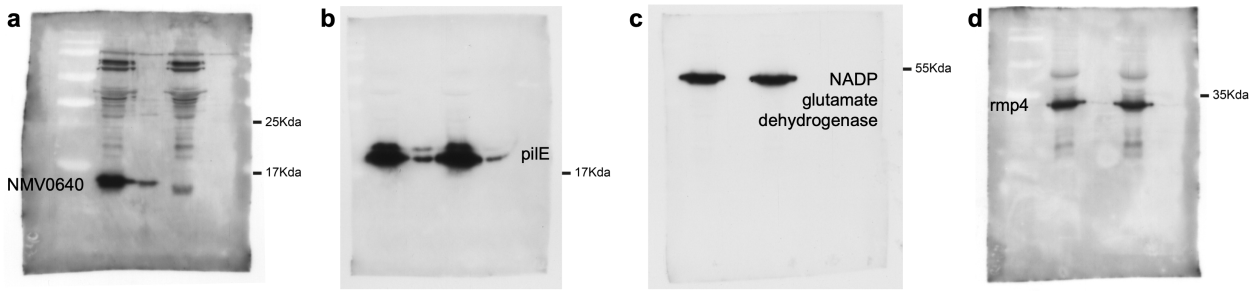

Supplementary Figure S3. Full-length blots of cropped blots shown in Figure 5 in the manuscript. a) NMV0640 (NMA1982). **b)** pilE. **c)** NADP glutamate dehydrogenase. **d)** Rmp4.

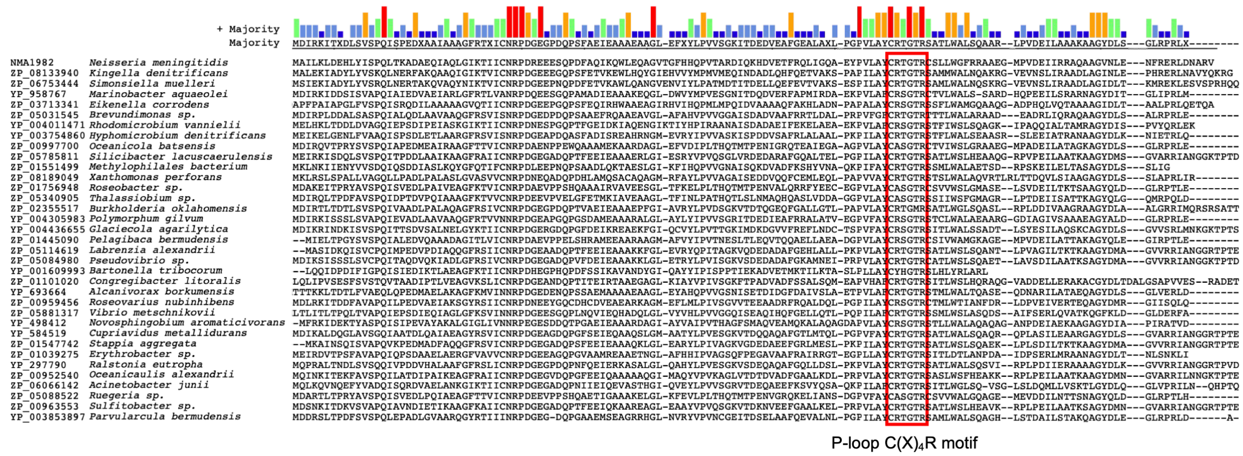

Supplementary Figure S4. Bacterial homologs of NMA1982. Clustal W alignment of NMA1982 and homologous protein sequences. Proteins other than NMA1982 are referred to by their NCBI Reference Sequence numbers. The ruler refers to NMA1982 residue numbers. The consensus (‘Majority’) and the consensus strength (‘+ Majority’) are given at the top. The color code of the consensus strength is red, 100% conserved, and dark blue, least conserved.

ELISA Test:

Lot.:11229	P1	P2	1000X	2000X	4000X	8000X	16000X	32000X	64000X	Normal 16000x	Normal 16000x	Normal 16000x	NC Average
His-conjugated NMA1982 protein (From Customer)	0.892	1.48	1.619	1.534	1.425	1.226	0.9	0.618	0.359	0.054	0.07	0.068	0.064
His tag Crude Extract	2.088	0.091	0.088	0.074	0.063	0.058	0.056	0.054	0.052	0.056	0.06	0.057	0.058

Lot.:11230	P1	P2	1000X	2000X	4000X	8000X	16000X	32000X	64000X	Normal 16000x	Normal 16000x	Normal 16000x	NC Average
His-conjugated NMA1982 protein (From Customer)	0.867	1.76	1.84	1.756	1.761	1.767	1.624	1.479	1.187	0.074	0.067	0.076	0.072
His tag Crude Extract	2.185	0.138	0.146	0.109	0.087	0.069	0.059	0.055	0.056	0.063	0.062	0.065	0.063

Coating : His-conjugated NMA1982 protein (From Customer) 0.2ug/well
His tag Crude Extract 1ul/well

Substrates : TMB
Exposure time: 5 mins
Absorbent : O.D 620

Western Blot:

Lot.: 11229 Lot.: 11230

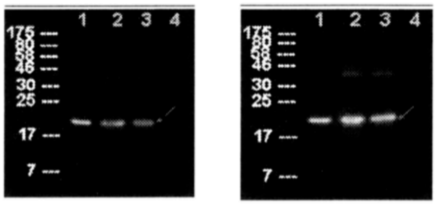

Loading : lane 1 ~4-- His-conjugated NMA1982 protein (From Customer)
0.2 ug/lane <MW : 17.5>

1st Ab : lane 1 -- Anti-His 2500x
lane 2 -- D56 Pre-bleed 1000x
lane 3 -- Unpurify polysera 5000x
lane 4 -- Normal sera 5000x

2nd Ab: lane 1 --Goat anti-mouse IgG-HRP(5000x)

Lot.: 11229 Lot.: 11230

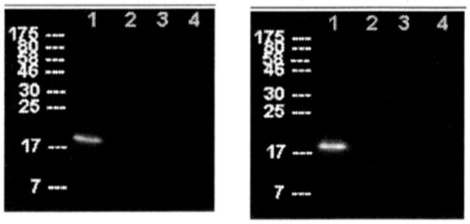

loading : lane 1 ~4-- His tag Crude Extract 1ul/lane

1st Ab : lane 1 -- Anti-His 2500x
lane 2 -- D56 Pre-bleed 1000x
lane 3 -- Unpurify polysera 5000x
lane 4 -- Normal sera 5000x

2nd Ab: lane 1 --Goat anti-mouse IgG-HRP(5000x)

Supplementary Figure S5. Performance of the polyclonal rabbit anti NMA1982 antibodies (Abnova, Taiwan). Antibody performance from two batches (Lot #11229 and Lot #11230) was tested against recombinant His-NMA1982 using both ELISA and immunoblot assays.
